# Validation of a Novel Virtual Reality Simulator for Robotic Surgery

**DOI:** 10.1155/2014/507076

**Published:** 2014-01-30

**Authors:** Henk W. R. Schreuder, Jan E. U. Persson, Richard G. H. Wolswijk, Ingmar Ihse, Marlies P. Schijven, René H. M. Verheijen

**Affiliations:** ^1^Division of Women and Baby, Department of Reproductive Medicine and Gynaecology, University Medical Centre Utrecht, P.O. Box 85500, Room F05-126, 3508 GA Utrecht, The Netherlands; ^2^Department of Obstetrics & Gynaecology, Skåne University Hospital, Tornavagen 10, 221 85 Lund, Sweden; ^3^Department of Surgery, Skåne University Hospital, Tornavagen 10, 221 85 Lund, Sweden; ^4^Lund Clinical Skills Center, Skåne University Hospital, Barngatan 2 B, 221 85 Lund, Sweden; ^5^Department of Surgery, Academic Medical Centre, P.O. Box 22660, 1100 DD Amsterdam, The Netherlands

## Abstract

*Objective*. With the increase in robotic-assisted laparoscopic surgery there is a concomitant rising demand for training methods. The objective was to establish face and construct validity of a novel virtual reality simulator (dV-Trainer, Mimic Technologies, Seattle, WA) for the use in training of robot-assisted surgery. *Methods*. A comparative cohort study was performed. Participants (*n* = 42) were divided into three groups according to their robotic experience. To determine construct validity, participants performed three different exercises twice. Performance parameters were measured. To determine face validity, participants filled in a questionnaire after completion of the exercises. *Results*. Experts outperformed novices in most of the measured parameters. The most discriminative parameters were “time to complete” and “economy of motion” (*P* < 0.001). The training capacity of the simulator was rated 4.6 ± 0.5 SD on a 5-point Likert scale. The realism of the simulator in general, visual graphics, movements of instruments, interaction with objects, and the depth perception were all rated as being realistic. The simulator is considered to be a very useful training tool for residents and medical specialist starting with robotic surgery. *Conclusions*. Face and construct validity for the dV-Trainer could be established. The virtual reality simulator is a useful tool for training robotic surgery.

## 1. **Introduction**

Since the FDA approval of the da Vinci Surgical System (dVSS) (Intuitive Surgical, Sunnyvale, CA) for gynaecological surgery, there has been an exponential growth in robot-assisted gynaecologic surgical procedures [1]. The field of robot-assisted minimal invasive surgery is still expanding and currently involves many specialties, including urology, general surgery, cardiothoracic surgery, paediatric surgery, and gynaecology. Current and future developments will further increase the uptake of robot-assisted procedures for minimal invasive surgery [2].

With the increase in robotic procedures, there is a concomitant rising demand for training methods for the dVSS. For laparoscopic surgery, the advantages of an ex vivo training program are well established. Laparoscopic skills can be learned using inanimate box/video trainers [3] and/or virtual reality (VR) trainers [4]. The acquired skills can be transferred to real operations, leading to a shorter operating time and less errors [5–7]. For robotic surgery, the development of training programs has just started and, like in laparoscopy, calls for competence-based training programs. Especially in technological highly advanced surgical methods, such as robotic-assisted surgery, surgeons should be properly trained before embarking on performing surgical procedures in patients. For robotic surgery, guidelines for training and credentialing were described in a consensus statement in 2007 [8]. Current robotic training programs for residents may involve dry labs with inanimate training, observation, bedside assisting and live surgery training [9, 10]. The main disadvantages of dry lab training are the lack of objective automated assessment, and extra non-cost-effective training on robotic systems is necessary to be able to familiarize with and master the robot system. To overcome these limitations, VR simulation could be the solution in training this new surgical technique before embarking robotic surgery in patients [11].

In 2010, a VR trainer for robotic surgery, the dV-Trainer (dVT) (Mimic, Technologies, Seattle, WA, USA), was introduced. During the development of the system, training on a prototype compared with training on the dVSS provided similar improvement of robotic skills on the dVSS [12]. This assumes VR training could be used for training robotic skills. Before the dVT can be implemented in a gynaecologic training program for robotic surgery, the first steps of the validation process (face and construct validity) must be established [13]. We designed a prospective study to establish face and construct validity of the dVT among a large group of gynaecologists. 

## 2. **Methods**

### 2.1. Participants

During the 2nd European Symposium on Robotic Gynaecologic Surgery, participants volunteering to the study were asked to complete three training modules on the VR simulator. The participants (*n* = 42) were categorized, according to their experience with robotic surgery (total amount of robotic cases performed), into three groups. Group 1 (*n* = 15), “novice,” had no experience in robotic surgery, Group 2 (*n* = 14), “intermediate,” performed more than 5 and less than 50 robotic cases, and Group 3 (*n* = 13), “expert,” performed more than 70 robotic cases. The novice group consisted of students, residents, and medical specialists. The intermediate and expert groups consisted of gynaecologic surgeons with varying robotic experience. Prior laparoscopic experience was stated as the number of level I-II laparoscopic procedures (diagnostic, sterilization, tubectomy, salpingectomy, or cystectomy) and the number of level III-IV laparoscopic procedures ((radical) hysterectomy, lymphadenectomy, or sacrocolpopexy). This was according to the guidelines of the European Society of Gynaecologic Endoscopy (ESGE) [14].

### 2.2. Equipment

**Figure 1:**
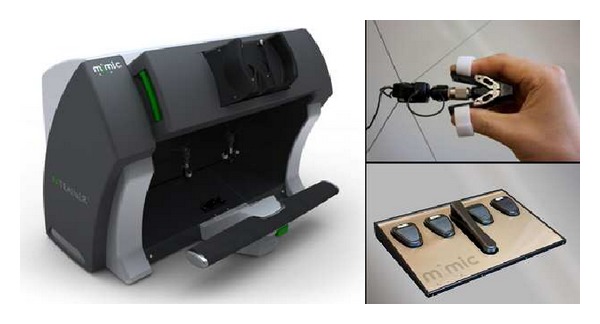
The dV-Trainer (showing console, grips, and pedals (image provided by Mimic Technologies, Inc., Seattle, WA)).

**Figure 2:**
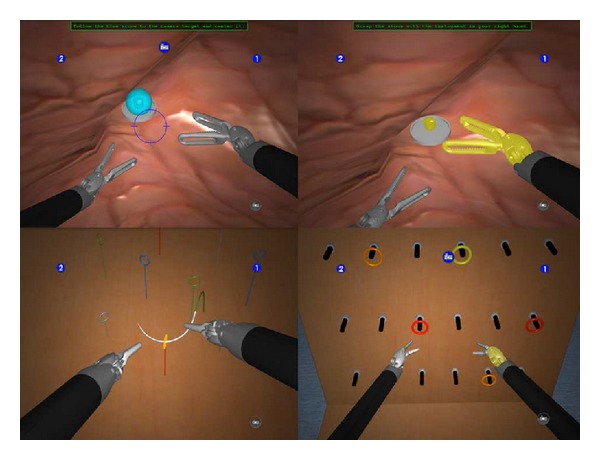
Exercises (exercises used in this study: “Camera Targeting,” “Thread the Rings,” and “Peg Board” (image provided by Mimic Technologies, Inc., Seattle, WA)).

The dVT is a VR simulator especially designed for training robotic surgery with the dVSS. This simulator consists of a two-handed haptic system with grips that emulate the master grips on the surgeon's console. Together with pedals and a high definition stereoscopic display, it simulates the console of the dVSS (Figure 1). The haptic component of the dVT includes a 580 MHz microprocessor and a 100 Mb Ethernet interface for data transfer and networking. The haptic device is networked with a computer that runs the dVT simulation software. The simulation system contains an automated system to measure different parameters of performance. The comprehensive training program of the dVT is subdivided in two sections. The “overview and basic skills training” consists of four modules: surgeons console overview, EndoWrist manipulation, camera and clutching, and trouble shooting. The “surgical skills training” includes the following four modules: needle control, needle driving, energy and dissection, and games. For this study we used the basic skill exercise “Camera Targeting,” the EndoWrist exercise “Peg Board,” and the surgical skill exercise “Thread the Rings” (Figure 2). All exercises have three levels of difficulty. For the purpose of this study we used the intermediate level (level 2) for all exercises.

### 2.3. Face Validity

Face validity is defined as the extent to which the simulator resembles the situation in the real world [13]. To investigate this, all participants filled out a questionnaire immediately after performing all three exercises. The first section of the questionnaire contained several questions about demographics, previous experience with VR trainers, laparoscopy, and robotic surgery. The second section contained 28 questions regarding the simulator, the exercises, and the training capacity of the simulator. These questions were used for establishing face validity and were presented on a 5-point Likert scale [15]. Finally, three general statements concerning training robotic surgery were made. These statements could be answered with “yes,” “no,” or “no opinion”.

### 2.4. Construct Validity

Construct validity is defined as the ability of the simulator to distinguish the experienced from the inexperienced surgeon [13]. To investigate construct validity, all participants were asked to perform each of the three exercises twice. Before starting on the simulator, the exercises were briefly explained by the test supervisor and introduced with an instruction video for each exercise. The first run of each exercise was used for familiarization with the simulator only, and verbal instructions were given whenever necessary. The second run on each exercise was used for validation purposes and data analysis. The first exercise was “Camera Targeting” (level 2), in which the goal of the exercise is to “accurately position the camera while manipulating objects in a large workspace.” The second exercise was “Thread the Rings” (level 2), in which the goal is to “develop accuracy when driving a needle and practice two-handed needle manipulation with hand offs between instruments.” The third and last exercise was “Peg Board” (level 2) in which the goal is to “improve EndoWrist dexterity and coordinated two-handed motion. Practice handling of objects between instruments and learn to avoid unwanted collisions of instruments with the surrounding environment and to develop camera control skills in the context of a pegboard task.” For all three exercises, the outcome parameters were measured during the second run of the participant. The outcome parameters and their definition are shown in Table 1.

### 2.5. Statistical Analysis

A sample size of on average 14 subjects per group allows for detection of between-group differences of 0.85 standard deviations (i.e., Cohen's *D* = 0.85) with 80% power, using alpha = 0.05 and assuming a correlation between scores on the same individual of 0.5. The collected data were analyzed using the statistical software package SPSS 15.0 (SPSS Inc, Chicago, IL). Differences between the group performances were analyzed using the Kruskal-Wallis test. If there appeared to be a significant difference, then a comparison between two separate groups was conducted using the Mann-Whitney *U* test for analysis of nonparametric data. A level of *P* ≤ 0.05 was considered to be statistically significant.

## 3. **Results**

### 3.1. Demographics

Forty-two subjects participated in this study. None of the participants had prior substantial experience with this simulator. Three participants, one in each group, had seen the simulator once before. There was no significant difference between the groups in prior experience with other VR simulators (*P* = 0.988). The prior laparoscopic experience of the intermediate and the expert groups was not significantly different for level I-II procedures (*P* = 0.756) and level III-IV procedures (*P* = 0.280). In the expert group, a wide range of total robotic cases was performed (70–1200). Demographics are shown in Table 2.

### 3.2. Face Validity

All participants completed the questionnaire. The mean scores regarding the simulator and the exercises are shown in Table 3. The realism of the simulator in general, visual graphics, movements of instruments, interaction with objects, and the depth perception were rated as realistic. The lowest score for the simulator in general was given for depth perception and for this item there was a significant difference between the novice group and intermediate group (*P* = 0.014). For all exercises, the participants stated that the goal of the exercise was surely reached. The training capacity of all separate exercises was rated to be “very good” by all groups. Compared to the novice group, the expert group rated the training capacity of “Thread the Rings” significantly higher (*P* = 0.041). The exercises were rated as “moderately difficult”; the “Peg Board” was considered to be the easiest exercise. The novice group and the intermediate group rated “Camera Targeting” significantly more difficult than the expert group (resp., *P* = 0.007 and *P* < 0.001). There were no other significant differences between the three groups.

The mean scores regarding training capacity in general are shown in Table 4. The training capacity of the simulator in general was rated “very good” (4.7 ± 0.5 SD). The training capacity for eye-hand coordination (4.5 ± 0.7 SD), camera navigation (4.5  ±  0.9), instrument navigation (4.4  ±  0.8 SD), and use of pedals and clutch (4.60 ± 0.8 SD) were all appreciated by the participants. The simulator was rated as a “very useful” training tool for junior residents, senior residents, and fellows or medical specialists starting with robotic surgery. The simulator was rated “moderately useful” for training robotic experts (3.3 ± 1.4 SD). The participants thought the simulator to be less useful for warmup before surgery or for retention of skills.

At the end of the questionnaire, three general statements about training robotic surgery were given. Almost everyone agreed on the statement that surgeons starting with robotics should first start training on a virtual system (no = 1, yes = 39, and no  opinion = 2). Most of the participants (86%) think it is time for a competence or proficiency based training curriculum for robotic surgery (no = 1, yes = 36, and no  opinion = 5). And the majority (74%) agreed that such a curriculum should be mandatory (no = 6, yes = 31, and no  opinion = 5). 

### 3.3. Construct Validity

**Figure 3:**
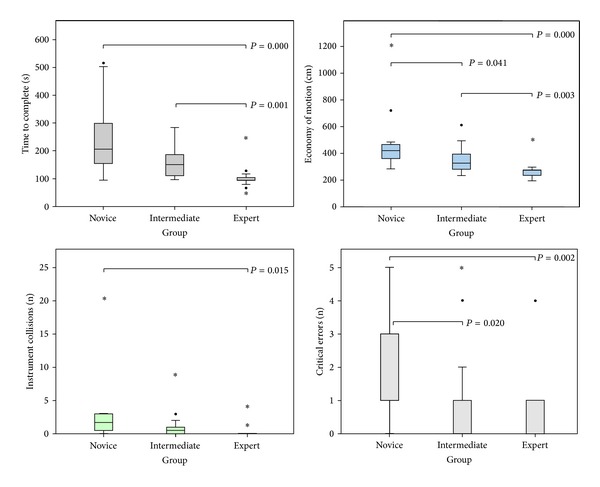
Exercise “Camera Targeting” (box plot of the four most important parameters in the exercise (bars are medians, boxes show inter quartile range, whiskers show range, ∙ are outliers, ∗ are extreme outliers, and large horizontal bars indicate statistically significant differences, specified with *P* values)).

**Figure 4:**
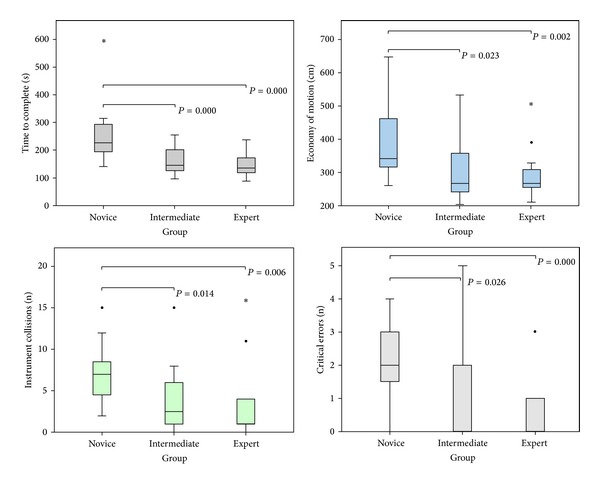
Exercise “Thread the Rings” (box plot of the four most important parameters in the exercise (bars are medians, boxes show inter quartile range, whiskers show range, ∙ are outliers, ∗ are extreme outliers, and large horizontal bars indicate statistically significant differences, specified with *P* values)).

All participants completed the three exercises on the dVT. For all parameters, the results and significant differences of the second run are shown in Table 5. Two important parameters showed a significant difference between all three groups; “economy of motion” in exercise 1 and “time to complete” in exercise 3. Comparison between the novice group and the expert group demonstrated the most significant differences. None of the other outcome parameters demonstrated a difference between novices and/or intermediates comparing more experienced colleagues. The performance variability of the most relevant parameters of the first two exercises are shown in box plots. The exercise “Camera Targeting” was most discriminative and showed significant differences in five parameters. There was less variability in the expert group (Figure 3). Four parameters showed significant difference in the exercise “Thread the Rings” (Figure 4). In the “Peg Board” exercise a significant difference was found in three parameters.

## 4. **Discussion**

In this study, the simulator showed good face validity. The dVT received high ratings on realism of the simulator itself and the separate exercises in all three groups. The training capacity of the simulator was rated “very good” for residents and gynaecologist starting with robotic surgery, but the simulator was found less useful for training experts. Perhaps the development of complex procedural tasks can add value for training experts who want to start performing new procedures. Using the dVT as warmup before surgery (to get familiar with the instruments again) or for retention of skills was not considered as real benefits of the simulator. Regarding the realism of the simulator, a remark should be made regarding the depth perception of the simulator. We noticed participants wearing multifocal glasses had a slight problem with the depth perception in the dVT. An explanation could be the difference in viewing distance in the dVT in contrast to length of this path in the dVSS. When participants changed their distance to the simulator/binoculars or did not wear their glasses during their performance, the problem regarding depth perception mainly declined. Unfortunately, in our questionnaire we did not ask participants if they wear glasses and therefore could not correlate this observation to results. 

The simulator was able to differentiate between novices and experts for a number of parameters in each exercise (construct validity). “Time to complete” the exercise and “economy of motion” were the two most discriminating parameters. For most parameters there was a significant difference between novices and experts, except for the “number of drops” and the distance of the “instruments out of view.” A possible cause for the nonsignificance in the “number of drops” may be due to the relatively easy level of difficulty, which limited the amount of drops in all exercises. According to the nonsignificance in “instrument(s) out of view,” all three groups had short periods of time in which instruments were out of view. However, the experts seemed to be not “loosing” their instruments, as intermediates and in particular novices did. For these less-experienced participants, this might be an explanation for the increased time to complete the exercises compared to their more experienced colleagues. There was less difference between the expert and the intermediate group. An explanation could be that even the level two exercises are still too easy to show a difference between these groups. This is supported by the fact that the most difficult exercise (Camera Targeting) showed a significant difference for “economy of motion” and “time to complete” between these two groups.

This is the first study which investigates the validity of the dVT in gynaecology. Previous several small studies in urology were performed during the beta development phase of the simulator, using relatively easy exercises [16–18]. Furthermore, the amount of participants (*n* = 42) was never as extensive as in this study and a comparison between three groups (novice, intermediate, and expert) was never conducted, since other studies only compared two groups (novice versus expert). The acceptability of the dVT was first addressed by Lendvay et al. In their survey, during a postgraduate training course in paediatric robotic surgery, the majority of participants believed that the dVT trainer could teach robotic skills comparable to a dry lab robotics skills station [19]. A study of 19 novices and seven experts validated a prototype of the dVT, demonstrating face and content validity of the simulator, but did not show construct validity [18]. In a similar study existing of a total of 15 participants with varying degree of urological experience, acceptability, and preliminary face and content validity was demonstrated. A positive correlation between robotic experience and key performance metrics was found. The authors concluded that more research is needed and suggested that a prospective study, similar in design to this study, could help to determine the utility to integrate this simulator into a robotic training curriculum [17]. Kenney et al. showed face, content, and construct validity for the dVT as a VR simulator for the dVSS. Nineteen novices and 7 experts completed two EndoWrist modules and two needle driving modules [16]. In our study, we found that the dVT was also able to distinguish between three groups of participants with different levels of robotic experience. The dVT simulator seems to provide almost equal training capacities compared with the real dVSS [12]. Moreover, training on the dVT can actually improve performance on the robot system equal to training with the robot itself. Improvement of technical surgical performance can be achieved within a relatively short period of time [20, 21]. Another important question is if this VR system could also be used for assessment of robotic skills [22]. Recently, Perrenot et al. concluded in their study that the dVT proves to be a valid tool to assess basic skills of robotic surgery on the dVSS [23].

Other groups are working on the development of different VR simulators for robotic surgery and reported about their prototypes [24, 25]. There is one laparoscopic simulator which can be converted into a robotic simulator and can train basic robotic skills [26]; however, van der Meijden et al. were not able to establish construct validity for this simulator and improvement is necessary before using it in robotic training programs [27]. Recently, face and content validity for another VR simulator (robotic surgical simulator (ROSS)) for robotic surgery was established [28, 29].

With the introduction of VR simulators for robotic surgery, a new tool for robotic training and credentialing has become available. Until now, most training programs for robotic surgery consist of didactics, hands-on dry lab training, instructional videos, assistance at the operating table, and performance of segments of an operation [30]. From there, some authors recommend to start with easy procedures to get familiar with the dVSS [31]. Virtual reality simulation could be of great value in robotic training programs and allow surgeons to develop skills and pass a substantial part of their learning curve before operating on humans [32]. The VR simulators provide a controlled and pressure free environment with real-time objective measurements of the trainees performance, thereby offering useful feedback for adequate self-assessment. This could eventually improve operating time and patient safety. The recommended way to use a VR simulator as a training tool is to implement it in a competence-based training curriculum [10]. Almost all of the participants in our study thought it is time for the development of competence-based training curricula for robotic surgery, this instead of the now often used time-based curricula. A vast majority of the participants even thought such training should be mandatory before starting robotic surgery. This is important since we know from laparoscopy that providing expensive simulators to trainees, without implementing them in an obligatory training curriculum, will not motivate them enough to train voluntarily [33, 34].

Recently, the dVT software exercises became commercially available for use directly on the da Vinci Si console with the release of the new da Vinci Skills Simulator. The hardware is attached to the actual robotic system as a separate box, or “backpack.” The box contains the software and can be used with the new Si robot models as an add-on tool. In this way, virtual training on the actual robotic console is possible. The first validation studies for the da Vinci Skills Simulator demonstrated good face, content, and construct validity [35, 36]. Recently, a prospective randomized study demonstrated the most ultimate forms of validity (concurrent and predictive validity) for the da Vinci Skills Simulator. In this study, the authors demonstrated that a simulator trained group actually performed better in real surgery on the dVSS [37]. In the future, the development of new modules will continue and complete VR procedures, like the hysterectomy, which will probably become available for use in the dVSS or on the stand-alone VR simulators.

## 5. ** Conclusions**

In conclusion, in this study, face and construct validity of the dVT was established. The simulator was regarded as a useful tool for training robotic surgery for the dVSS. For optimal use, the simulator should be implemented in validated competence-based robotic training curricula. Further studies regarding predictive validity need to show if simulator-learned skills are transferable to actual operations in the short run, and if so, whether or not positive effects on surgical performance remain on the long run.

## Figures and Tables

**Table 1:** Parameter definition.

Exercise and parameters	Definition
Errors (*n*)	If a user receives a 0% score for any metric, this is counted as an *n* error.
Drops (*n*)	“Camera Targeting”: number of times a stone is dropped outside of a basket or platform and hits the cavity wall. “Tread the Rings”: number of times the needle is dropped on the floor. “Peg Board”: number of times a ring is dropped in the floor.
Economy of motion (cm)	Total distance travelled by all EndoWrist tools; measured from the clevis, not the tool tips.
Excessive instrument force (sec)	Total time an applied instrument force exceeds a given force threshold.
Instrument collisions (*n*)	Number of times one instruments collides with another instrument.
Instrument(s) out of view (cm)	Total distance travelled by all instrument when not in view.
Master workspace range (cm)	Combined radius from two spheres that encapsulate the path travelled by the master grips.
Time to complete (sec)	Total time that begins when the user enters following mode and then ends when the user finishes or exits an exercise.

**Table 2:** Demographics.

Total (*n* = 42)	Group 1 Novice (*n* = 15)	Group 2 Intermediate (*n* = 14)	Group 3 Expert (*n* = 13)
Mean age (range)	39 (28–55)	44 (31–63)	48 (35–61)
Gender (male/female)	11/4	11/3	13/0
Participants (*n*)			
Medical specialist	3	12	13
Resident	3	2	0
Other	9	0	0
Videogame experience, >10 hours (no/yes)	7/8	9/5	11/2
Laparoscopic experience (level I-II procedures)			
0	10	0	0
1–25	2	1	0
26–100	1	1	3
>100	2	12	10
Laparoscopic experience (level III-IV procedures)			
0	11	2	0
1–24	2	0	0
25–100	2	7	6
>100	0	5	7
Robotic experience (total cases)			
None	15	0	0
5–9	0	3	0
10–39	0	6	0
40–49	0	5	0
70–150	0	0	9
>150	0	0	4
Mean estimated total number of robotic cases (range)	0	24 (6–50)	240 (70–1200)

**Table 3:** Face validity (simulator and exercises).

Questions	Group 1 Novice (*n* = 15)	Group 2 Intermediate (*n* = 14)	Group 3 Expert (*n* = 13)	Mean (*n* = 42)
*Simulator in general *				
What do you think of the realism of the next issues? (1 = very unrealistic…5 = very realistic)				
Simulator itself (hardware)	3.8 ± 0.8	4.2 ± 0.7	4.2 ± 0.6	4.1 ± 0.7
Visual graphics	4.3 ± 0.5	4.4 ± 0.7	4.0 ± 1.1	4.3 ± 0.8
Movements of the instruments	3.8 ± 0.9	4.4 ± 0.7	4.2 ± 0.7	4.1 ± 0.8
Interaction with objects	3.9 ± 0.9	4.2 ± 0.7	4.4 ± 0.8	4.2 ± 0.8
Depth perception	3.7 ± 0.9	4.6 ± 0.5	3.7 ± 1.4	4.0 ± 1.1
*Exercise 1 “Camera Targeting” *				
Do you think the goal of the exercise is reached? (1 = surely not…5 = surely yes)	4.5 ± 0.5	4.57 ± 0.5	4.4 ± 0.5	4.5 ± 0.5
What is your opinion according to the following issues? (1 = very bad…5 = very good)				
Content of the exercise	4.4 ± 0.6	4.2 ± 0.9	4.4 ± 0.8	4.3 ± 0.8
Training capacity of the exercise	4.5 ± 0.6	4.4 ± 0.8	4.5 ± 0.7	4.5 ± 0.7
Difficulty of the exercise	3.3 ± 1.0	3.6 ± 0.8	2.0 ± 1.1	3.0 ± 1.2
*Exercise 2 “Thread the Rings” *				
Do you think the goal of the exercise is reached? (1 = surely not…5 = surely yes)	4.3 ± 1.0	4.2 ± 0.89	4.6 ± 0.7	4.4 ± 0.9
What is your opinion according to the following issues? (1 = very bad…5 = very good)				
Content of the exercise	4.5 ± 0.6	4.5 ± 0.7	4.5 ± 0.7	4.5 ± 0.6
Training capacity of the exercise	4.3 ± 0.6	4.6 ± 0.6	4.9 ± 0.4	4.6 ± 0.6
Difficulty of the exercise	3.5 ± 0.9	3.3 ± 1.1	3.0 ± 1.0	3.3 ± 1.0
*Exercise 3 “Peg Board” *				
Do you think the goal of the exercise is reached? (1 = surely not…5 = surely yes)	4.3 ± 0.9	4.2 ± 0.58	4.6 ± 0.5	4.4 ± 0.7
What is your opinion according to the following issues? (1 = very bad…5 = very good)				
Content of the exercise	4.4 ± 0.7	4.2 ± 0.6	4.4 ± 0.7	4.3 ± 0.7
Training capacity of the exercise	4.3 ± 0.8	4.3 ± 0.8	4.5 ± 0.7	4.4 ± 0.8
Difficulty of the exercise	2.9 ± 0.9	2.9 ± 0.9	2.5 ± 1.2	2.7 ± 0.9

Values expressed in mean on a 1 to 5 Likert scale ± SD.

**Table 4:** Face validity (training capacity).

Questions	Group 1 Novice (*n* = 15)	Group 2 Intermediate (*n* = 14)	Group 3 Expert (*n* = 13)	Mean (*n* = 42)
Training capacity simulator in general (1 = very bad…5 = very good)	4.7 ± 0.5	4.8 ± 0.4	4.5 ± 0.5	4.6 ± 0.5
Training capacity simulator regarding (1 = very bad…5 = very good)				
Eye-hand coordination	4.5 ± 0.5	4.5 ± 0.9	4.5 ± 0.7	4.5 ± 0.7
Camera navigation	4.3 ± 1.1	4.5 ± 0.7	4.6 ± 0.9	4.6 ± 0.9
Instrument navigation	4.1 ± 1.1	4.6 ± 0.5	4.5 ± 0.5	4.4 ± 0.8
Use of pedals and clutch	4.5 ± 1.1	4.6 ± 0.6	4.7 ± 0.5	4.6 ± 0.8
The simulator is useful for training (1 = very unuseful…5 = very useful)				
Junior residents (postgraduate year 1–3)	4.5 ± 1.1	4.5 ± 1.2	4.9 ± 0.3	4.6 ± 0.9
Senior residents (postgraduate year 4–6)	4.5 ± 0.6	4.4 ± 1.2	4.6 ± 0.8	4.5 ± 0.9
Fellow's and regular consultants (starting robotics)	4.5 ± 0.7	4.9 ± 0.4	4.7 ± 0.6	4.7 ± 0.6
Robotic experts	3.2 ± 1.3	3.4 ± 1.3	3.2 ± 1.5	3.3 ± 1.4
The simulator is useful for (1 = very un useful…5 = very useful)				
Warmup for robotic surgery	3.9 ± 1.3	3.6 ± 1.5	2.7 ± 1.5	3.4 ± 1.4
Retention of robotic skills	3.8 ± 1.1	4.0 ± 1.1	2.9 ± 1.6	3.6 ± 1.3

Values expressed in mean on a 1 to 5 Likert scale ± SD.

**Table 5:** Construct validity.

Exercise and parameters	Group 1 Novice (*n* = 15)	Group 2 Intermediate (*n* = 14)	Group 3 Expert (*n* = 13)	Significant difference (*P* < 0.05)
Exercise 1 “Camera Targeting”				
Errors (*n*)	2.33 ± 1.45	1.00 ± 1.62	0.54 ± 1.11	1 > 2 (*P* = 0.020), 1 > 3 (*P* = 0.002)
Drops (*n*)	0.20 ± 0.41	0.00 ± 0.00	0.00 ± 0.00	
Economy of motion (cm)	469.74 ± 234.53	353.78 ± 99.20	268.21 ± 69.69	1 > 2 (*P* = 0.041), 1 > 3 (*P* < 0.001), 2 > 3 (*P* = 0.003)
Excessive instrument force (sec)	22.10 ± 36.92	11.36 ± 24.26	8.42 ± 29.48	
Instrument collisions (*n*)	2.87 ± 5.17	1.21 ± 2.16	0.46 ± 1.13	1 > 3 (*P* = 0.015)
Instrument(s) out of view (cm)	27.58 ± 42.76	10.06 ± 23.67	18.04 ± 47.49	
Master workspace range (cm)	12.65 ± 2.11	11.72 ± 2.02	10.61 ± 1.62	1 > 3 (*P* = 0.015)
Time to complete (sec)	246.27 ± 131.58	164.59 ± 64.24	106.60 ± 44.59	1 > 3 (*P* < 0.001), 2 > 3 (*P* = 0.001)
Exercise 2 “Thread the Rings”				
Errors (*n*)	2.13 ± 1.13	1.07 ± 1.54	0.46 ± 0.88	1 > 2 (*P* = 0.026), 1 > 3 (*P* < 0.001)
Drops (*n*)	0.40 ± 0.83	0.29 ± 0.61	0.38 ± 0.65	
Economy of motion (cm)	404.60 ± 120.98	308.03 ± 101.40	292.28 ± 74.42	1 > 2 (*P* = 0.023), 1 > 3 (*P* = 0.002)
Excessive instrument force (sec)	9.67 ± 13.15	5.50 ± 8.52	3.69 ± 4.20	
Instrument collisions (*n*)	6.93 ± 3.54	3.86 ± 4.15	3.38 ± 4.81	1 > 2 (*P* = 0.014), 1 > 3 (*P* = 0.006)
Instrument(s) out of view (cm)	2.04 ± 3.35	3.34 ± 9.18	0.74 ± 1.30	
Master workspace range (cm)	9.08 ± 1.97	8.46 ± 1.77	8.27 ± 1.24	
Time to complete (sec)	256.76 ± 107.70	158.17 ± 45.39	146.15 ± 39.65	1 > 2 (*P* < 0.001), 1 > 3 (*P* < 0.001)
Exercise 3 “Peg Board”				
Errors (*n*)	0.40 ± 0.63	0.14 ± 0.36	0.15 ± 0.38	
Drops (*n*)	0.73 ± 0.70	0.36 ± 0.50	0.15 ± 0.38	1 > 3 (*P* = 0.037)
Economy of motion (cm)	400.13 ± 109.58	342.35 ± 71.12	293.20 ± 73.08	1 > 3 (*P* = 0.002), 2 > 3 (*P* = 0.025)
Excessive instrument force (sec)	1.27 ± 3.09	0.39 ± 0.99	0.12 ± 0.30	
Instrument collisions (*n*)	1.13 ± 1.36	0.71 ± 0.83	0.46 ± 0.66	
Instrument(s) out of view (cm)	0.82 ± 2.25	0.37 ± 1.08	1.25 ± 3.40	
Master workspace Range (cm)	10.91 ± 1.93	9.51 ± 1.57	9.66 ± 1.51	
Time to complete (sec)	157.45 ± 44.05	114.46 ± 23.80	90.08 ± 26.72	1 > 2 (*P* = 0.007), 1 > 3 (*P* < 0.001), 2 > 3 (*P* = 0.017)

Values expressed in mean ± SD.
